# Reversal of pentylenetetrazole-induced seizure activity in mice by nickel chloride

**DOI:** 10.4103/0253-7613.48885

**Published:** 2009-02

**Authors:** Ashish K. Rehni, Nirmal Singh

**Affiliations:** Department of Pharmaceutical Sciences and Drug Research, Punjabi University, Patiala - 147 002, India

**Keywords:** Nickel, pentylenetetrazole, seizure

## Abstract

**Objective:**

The present study was designed to investigate the anticonvulsant potential of nickel which is shown to selectively block t-type calcium channels by using nickel choride on pentylenetetrazole (80 mg/kg) induced seizure activity model in mice.

**Materials and Methods:**

Seizures were assessed in terms of onset of Straub's tail phenomenon and onset of jerky movements of the whole body, convulsions, and death. Sodium valproate served as a standard control in the present study.

**Results:**

Nickel chloride (5 mg/kg i.p. and 10 mg/kg i.p.) attenuated pentylenetetrazole-induced seizure activity in mice, as reflected by a significant increase in the onset time of Straub's tail phenomenon and onset of jerky movements of the whole body, convulsions, and death. High dose of nickel chloride showed more pronounced anticonvulsant action than sodium valproate.

**Conclusions:**

The anticonvulsant action of nickel chloride was noticeable in this study. However, further studies are required to elucidate its full anticonvulsant potential.

## Introduction

Molecular cloning studies have revealed that heterogeneity of t-type Ca^2+^ currents in native tissues arises from the three isoforms of Cav3 channels: Cav3.1, Cav3.2, and Cav3.3.[1] From pharmacological analysis of the recombinant t-type channels, low concentrations (*<*50 μM) of nickel were found to selectively block the Cav3.2 over the other isoforms.[1] Nickel has been used to selectively block t-type currents in a number of cell types, such as sinoatrial nodal cells[1,2] and sensory neurons.[3] However, t-type currents in various neuronal cells require much higher doses of nickel to be blocked.[3,4] Pharmacological studies have shown that low-voltage-activated t-type Ca^2+^ channels are involved in the genesis of absence seizures.[5,6] Drugs that act by inhibiting neuronal t-type calcium currents like sodium valproate have potential activity against absence seizure.[7,8] Therefore, the present study was designed to evaluate the anticonvulsant potential of nickel, by using nickel chloride on pentylenetetrazole-induced seizure activity in mice.

## Materials and Methods

Male inbred BALB/C mice – each weighing 25 ± 2 g, maintained on standard laboratory diet (Kisan Feeds Ltd., Mumbai, India), and having free access to tap water – were employed in the present study. They were housed in the departmental animal house and were exposed to 12-hour light–dark cycle. The protocol of study was approved by animal ethics committee of the department and the experiments were carried out as per the guidelines of committee for the purpose of control and supervision of experiments on animals (CPCSEA), Ministry of Environment and Forest Government of India.

### Induction of seizures

Seizure activity was induced in wakeful mouse using an intraperitoneal (i.p.) injection of pentylenetetrazole (80 mg/kg). Time of appearance of Straub's tail andonset of jerky movements of the whole body, and convulsions were recorded as a measure of the severity of experimental epileptic activity elicited by the administration of the drug.[9] Mortality percentage of animals post pentylenetetrazole administration in various treatment groups was recorded and the data were employed to evaluate ED_50_ value of nickel chloride as well as sodium valproate.

### Experimental protocol

In the present study, five groups were employed and each group comprised 10 animals.

- Group-I (pentylenetetrazole-treated control group): Mice were administered pentylenetetrazole (80 mg/kg, i.p.).
- Group-II (vehicle + pentylenetetrazole-treated control group): Mice were administered vehicle (10 ml/kg, i.p.) 30 min prior to the injection of pentylenetetrazole (80 mg/kg, i.p.).
- Group-III (sodium valproate + pentylenetetrazole-treated standard control group): Animals were administered sodium valproate (150 mg/kg, i.p.) 30 min prior to the injection of pentylenetetrazole (80 mg/kg, i.p.).
- Group-IV (low-dose nickel + pentylenetetrazole treatment group): Mice were administered nickel chloride (5 mg/kg, i.p.) 30 min prior to the injection of pentylenetetrazole (80 mg/kg, i.p.).
- Group-V (High-dose nickel + pentylenetetrazole treatment group): Animals were administered nickel chloride (10 mg/kg, i.p.) 30 min prior to the injection of pentylenetetrazole (80 mg/kg, i.p.)

### Drugs and chemicals

Pentylenetetrazole (Sigma, St. Louis, USA), sodium valproate (Sun Pharma, Mumbai, India), and nickel chloride (Central Drugs House (P) Ltd., New Delhi, India) were dissolved in normal saline. All drug solutions were freshly prepared before use.

### Statistical analysis

Data obtained from the study were statistically analyzed using one-way ANOVA followed by Tukey's multiple range test as *post-hoc* analysis. Statistical analysis for the results of mortality was done using chi-square test. A value of *P* < 0.05 was considered to be statistically significant.

## Results

The ED_50_ values, calculated based on the percentage mortality of animals post pentylenetetrazole administration in various treatment groups, were 2.28 mg/kg and 53 mg/kg for nickel chloride and sodium valproate, respectively.

### Effect of pentylenetetrazole, sodium valproate, and nickel chloride on the onset time Straub's tail phenomenon

**Figure 1:**
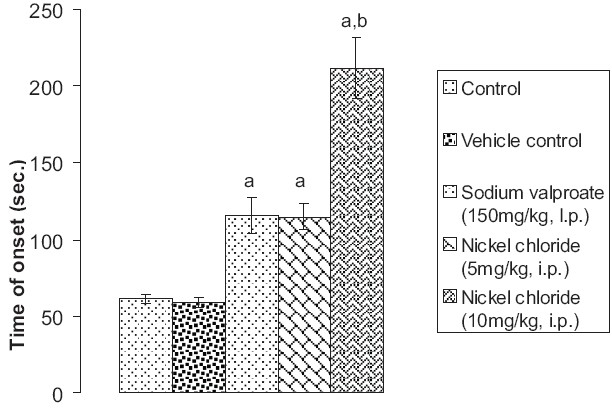
Effect of sodium valproate and nickel chloride on pentylenetetrazole-induced seizures in mice as assessed in terms of time of onset of Straub's tail phenomenon Values are expressed as mean ± SEM. Statistical analysis for the results was done using one-way ANOVA followed by Tukey's multiple range test as post-hoc analysis a = *P* < 0.05 vs control; b = *P* < 0.05 vs sodium valproate

Although vehicle did not have any effect, prior administration of sodium valproate (150 mg/kg, i.p.) significantly (*P* < 0.05) attenuated pentylenetetrazole-induced seizure activity in mice in terms of onset time of Straub's tail phenomenon. Prior administration of nickel chloride (5 mg/kg i.p. and 10 mg/kg i.p.) also significantly (*P* < 0.05 and *P* < 0.01) attenuated pentylenetetrazole-induced seizure activity in mice measured in terms of onset time of Straub's tail phenomenon. However, the extent of delay in the onset time of Straub's tail phenomenon induced by the high-dose nickel chloride (group V) was found to be significantly more marked (*P* < 0.01) than by sodium valproate (*P* < 0.05) [Figure 1].

### Effect of pentylenetetrazole, sodium valproate, and nickel chloride on onset time of jerky movements of the whole body

**Figure 2:**
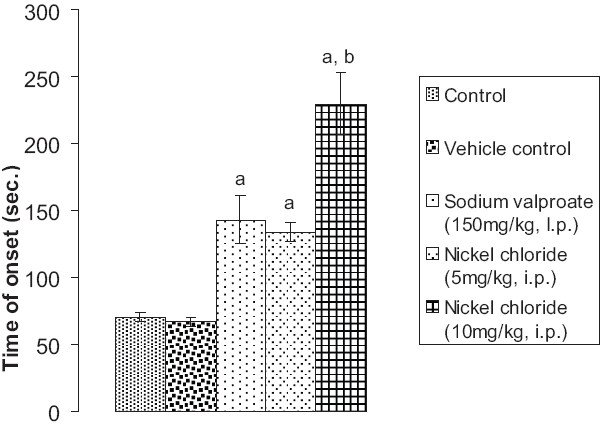
Effect of sodium valproate and nickel chloride on pentylenetetrazole-induced seizures in mice as assessed in terms of time of onset of jerky movements of the whole body Values are expressed as mean ± SEM. Statistical analysis for the results was done using one-way ANOVA followed by Tukey's multiple range test as post-hoc analysis a = *P* < 0.05 vs control; b = *P* < 0.05 vs sodium valproate

Although vehicle did not have any effect, prior administration of sodium valproate (150 mg/kg, i.p.) significantly prevented pentylenetetrazole-induced seizure activity in mice in terms of onset time of jerky movements of the whole body. Prior administration of nickel chloride (5 mg/kg i.p. and 10 mg/kg i.p.) also significantly (*P* < 0.05 and *P* < 0.01) attenuated pentylenetetrazole-induced seizure activity in mice measured in terms of onset time of jerky movements of the whole body. However, the extent of delay in the onset time of jerky movements of the whole body induced by the high-dose nickel chloride (group V) was found to be significantly more noticeable (*P* < 0.01) than by sodium valproate (*P* < 0.05) [Figure 2].

### Effect of pentylenetetrazole, sodium valproate, and nickel chloride on onset time of convulsions

**Figure 3:**
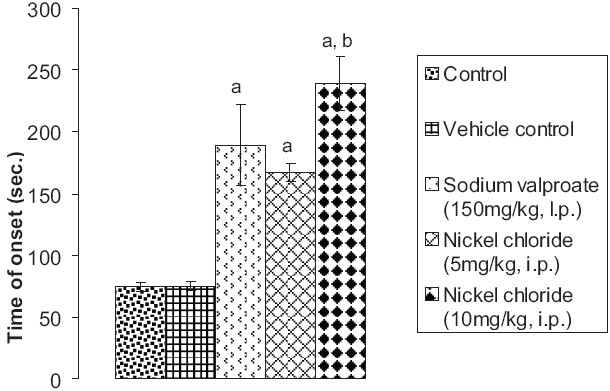
Effect of sodium valproate and nickel chloride on pentylenetetrazole-induced seizures in mice as assessed in terms of time of onset of convulsions Values are expressed as mean ± SEM. Statistical analysis for the results was done using one-way ANOVA followed by Tukey's multiple range test as post-hoc analysis

Although vehicle did not have any effect, prior administration of sodium valproate (150 mg/kg, i.p.) significantly decreased pentylenetetrazole-induced seizure activity in mice in terms of onset time of convulsions. Prior administration of nickel chloride (5 mg/kg i.p. and 10 mg/kg i.p.) also significantly attenuated pentylenetetrazole-induced seizure activity in mice measured in terms of onset time of convulsions. Moreover, the extent of delay in the onset time of convulsions induced by the high-dose nickel chloride (*P* < 0.01) treatment group was found to be significantly more pronounced than by sodium valproate (*P* < 0.05) [Figure 3].

### Effect of pentylenetetrazole, sodium valproate, and nickel chloride on percentage mortality in mice

**Figure 4:**
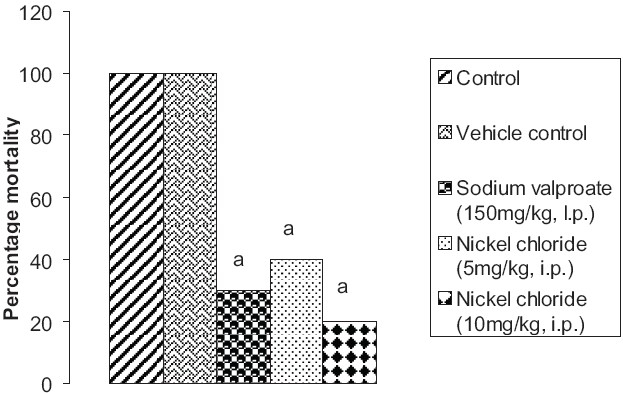
Effect of sodium valproate and nickel chloride on pentylenetetrazole-induced mortality in mice Values are expressed as percentage mortality. Statistical analysis for the results of mortality was done using chi square test a = *P* < 0.05 vs control; b = *P* < 0.05 vs sodium valproate

Administration of pentylenetetrazole induced a significant increase (*P* < 0.05) in percentage mortality of mice. Although vehicle did not have any effect, prior administration of sodium valproate (150 mg/kg, i.p.) as well as nickel chloride (5 mg/kg i.p. and 10 mg/kg i.p.) significantly (*P* < 0.05) reversed the mortality rate [Figure 4].

## Discussion

The administration of pentylenetetrazole in the present study induced Straub's tail phenomenon, followed by jerky movements of the whole body, and convulsions in pentylenetetrazole-treated control group animals along with an increase in the percentage mortality of mice. Pentylenetetrazole is a chemoconvulsant, which induces seizures by the inhibition of GABAA receptors and is widely accepted experimental model for absence seizure.[7,8] These observations are in line with the previous findings.[9] Administration of sodium valproate markedly attenuated pentylenetetrazole-induced seizure activity in mice observed in terms of onset time of Straub's tail, jerky movements of the whole body, as well as convulsions. In addition, there was a significant decrease in percentage mortality of animals. Sodium valproate has been shown to be an effective agent in ameliorating the symptoms of generalized absence epilepsy via blockade of voltage dependent t-type calcium channels.[10–11] Thus, our results are in consonance with previous reports and sodium valproate served as a standard control in the present study.

Nickel chloride inhibited in a significant manner, pentylenetetrazole-induced seizures as assessed in terms of time of appearance of Straub's tail and onset of jerky movements of whole body and convulsions. Moreover, there was a significant reduction in percentage mortality of mice. Molecular cloning studies have revealed that heterogeneity of t-type Ca^2+^ currents in native tissues arises from the three isoforms of Cav3 channels: Cav3.1, Cav3.2, and Cav3.3.[1] From pharmacological analysis of the recombinant t-type channels, low concentrations (<50 *μ*M) of nickel were found to selectively block the Cav3.2 over the other isoforms.[1–4] The Cav3.2 isoform of t-type calcium channels is widely distributed in the CNS and have been shown to be involved in mediating the effect of nickel.[12] Therefore, it may be suggested that nickel-induced blockade of T-type calcium channels may be involved in the reduction of seizures elicited by pentylenetetrazole. However, what accounted for more pronounced effect of nickel than sodium valproate remains to be elucidated. Nevertheless, further studies are required to unearth full potential of nickel chloride as an anticonvulsant.
